# *Neisseria macacae* bacteremia: report of two cases with a literature review

**DOI:** 10.1186/s12879-020-05346-3

**Published:** 2020-08-24

**Authors:** Yasumasa Yamamoto, Norihiko Terada, Tomoyo Sugiyama, Hanako Kurai, Kiyofumi Ohkusu

**Affiliations:** 1 Division of Infectious Diseases, Shizuoka Cancer Center Hospital, 1007 Shimonagakubo, Sunto-gun, Shizuoka, Nagaizumi, 411-8777 Japan; 2 Department of Microbiology Tokyo Medical University, Shinjyuku, Tokyo, Japan

**Keywords:** *Neisseria macacae*, *Neisseria* species, Bacteremia, Cancer

## Abstract

**Background:**

*Neisseria macacae* was discovered in the oral cavity of monkeys in 1983. In humans, it has been isolated from the upper respiratory tract of neutropenic patients. However, only two cases of *N. macacae* bacteremia have been reported in a 65-year-old man with infective endocarditis and a 5-month-old child with fever and petechiae. There are no reports of infections in cancer patients. Here, we present two cases of *N. macacae* bacteremia in cancer patients.

**Case presentation:**

In the first case, a 42-year-old woman who underwent ovarian cancer surgery presented with duodenal invasion associated with multiple lymph node metastasis. *N. macacae* was isolated from her blood culture and identified using matrix-assisted laser desorption/ionization time-of-flight (MALDI-TOF) mass spectrometry (MS). In the second case, a 69-year-old woman with a long-standing history of esophagogastric junction cancer presented with fever. She had stage IVB cancer with lung, bone, and multiple lymph node metastasis. The last chemotherapy was administered 5 weeks before *N. macacae* was detected using MALDI-TOF MS and nitrate test negative. In both cases, transthoracic echography showed no vegetation. Antibiotics were administered for 14 and 13 days in the first and second cases, respectively. In both cases, fever alleviated on day 4 of antibiotic administration. Both patients were discharged after their conditions improved.

**Conclusions:**

This, to our knowledge, is the first report of *N. macacae* bacteremia in cancer patients. Both patients, mucosal damage was observed in the upper gastrointestinal tract. Therefore, exclusion diagnosis suggested that bacteremia invasion was caused by mucosal rupture in both cases. Both cases responded well to treatment with β-lactam antibiotics and improved after 2 weeks. Modifying the treatment based on the source of the infection may shorten the treatment period. Therefore, further research on *N. macacae* bacteremia is necessary. Immunocompromised patients such as those with cancer are susceptible to mucosal damage by unusual bacterial species such as *N. macacae* despite not having contact with monkeys.

## Background

*Neisseria macacae* is a *Neisseria* species that was discovered in the oral cavity of monkeys in 1983. In humans, it has been isolated from the upper respiratory tract of neutropenic patients. Human infections of *N. macacae* are extremely rare. We found only two previous cases of *N. macacae* bacteremia in humans. Previously reported cases included a 65-year-old man with infective endocarditis and a 5-month-old child with fever and petechiae. To the best of our knowledge, there are no reports of infections in patients with cancer. Depending on the condition of the patients with cancer, this infection may be more likely to occur. This is the first report of two cases of *N. macacae* bacteremia in adult cancer patients. In both cases, *N. macacae* was identified using matrix-assisted laser desorption/ionization time-of-flight (MALDI-TOF) mass spectrometry (MS) and 16S RNA analysis.

## Case presentation

### Case 1

A 42-year-old woman with a long-standing history of ovarian cancer presented with fever. She had stage IVB cancer with multiple lymph node metastasis and peritoneal dissemination. Abdominal total hysterectomy, bilateral salpingo-oophorectomy, and omentectomy were performed 1 year ago. The last chemotherapy was performed 3 months ago. The patient denied any contact with monkeys. On examination, she appeared unwell and was experiencing intense rigors. Her temperature was 38.3 °C, heart rate was 95 beats/min and regular, blood pressure was 102/61 mmHg, respiratory rate 18 breaths/min, and oxygen saturation was 96% in room air. Laboratory investigations revealed an elevated white blood cell count of 8770/μL (neutrophil count 6840/μL), microcytic anemia (hemoglobin level 9.3 g/dL), C-reactive protein (CRP) 19.99 mg/dL (normal < 0.30 mg/dL), and persistently elevated cholestatic liver enzyme levels (gamma glutamyltransferase 78 U/L [normal < 30 U/L and alkaline phosphatase 575 U/L [normal 115–359 U/L]). Mild tenderness in the upper abdomen was observed. Blood and urine cultures were performed. No significant bacteria were detected in the urine cultures, including *Neisseria*. A computed tomography scan of the patient revealed duodenal invasion with enlarged lymph nodes. Empirical antimicrobial therapy comprising intravenous piperacillin-tazobactam at 4.5 g was administered every 6 h. Only the aerobic culture showed bacterial growth 34 h after starting culture using BD resin bottles (Becton Dickinson, Tokyo, Japan) and the BD Bactec™ FX blood culture system (Becton Dickinson, Tokyo, Japan). Subsequently, gram-negative diplococcus was identified and confirmed as *N. macacae* using MALDI-TOF MS (Biotyper:BRUKER Score Value 2.10) (Table 1). No other organisms were detected in the blood culture. Broth microdilution method (DP34, Eiken, Japan) showed that the bacterial culture was susceptible to ceftriaxone, ampicillin, meropenem, and minocycline but resistant to penicillin, trimethoprim-sulfamethoxazole, and levofloxacin (Clinical Laboratory Standards Institute [CLSI] testing methods and MIC break point for *N. meningitidis* was substituted). Transthoracic echography showed no vegetations. Fever alleviated by day 4 of antimicrobial administration. On day 6, her treatment was changed to intravenous ampicillin-sulbactam 3 g every 6 h. The total antibiotic treatment period was 14 days. Her condition improved, and she was discharged. After discharge, she was followed up for 1 month in an outpatient setting, and there was no recurrence.

**Table 1:** Case 1: MALDI-TOF MS results

Rank	Matched Pattern	Score Value
1	*Neisseria macacae* DSM19175T	2.05
2	*Neisseria flavescens* C12PGM	1.89
3	*Neisseria mucosa* DSM17611T	1.74
4	*Neisseria mucosa* 1591PGM	1.70
5	*Neisseria lactamica* VA33589	1.67
6	*Neisseria sp* 0807 M15057901 IBS	1.65
7	*Neisseria flavescens* DSM17633T	1.58
8	*Neisseria sicca* DSM17713T	1.56
9	*Neisseria perflava* DSM18009T	1.55
10	*Neisseria subflava* DSM17610T	1.53

### Case 2

A 69-year-old woman with a long-standing history of esophagogastric junction cancer presented with fever. She had stage IVB cancer with lung, bone, and multiple lymph node metastasis. The last chemotherapy was performed 4 weeks ago. The patient denied any contact with monkeys. On examination, she appeared unwell and was experiencing intense rigors. Her temperature was 37.8 °C, heart rate was 100 beats/min and regular, blood pressure was 137/82 mmHg, respiratory rate was 18 breaths/min, and oxygen saturation 99% in room air. Laboratory investigations revealed an elevated white blood cell count of 5450/μL (neutrophil count 4180/μL), microcytic anemia (hemoglobin level 9.8 g/dL), CRP 0.64 mg/dL [normal < 0.30 mg/dL], and persistently elevated gamma glutamyltransferase 40 U/L [normal < 30 U/L]. Mild tenderness in the upper abdomen was observed. Blood and urine cultures were performed. No significant bacteria were detected in the urine cultures, including *Neisseria*. A computed tomography scan of her abdomen was performed which revealed esophagogastric junction cancer and multiple lymph node metastatic lesions which were enlarged. Empirical antimicrobial therapy comprising intravenous piperacillin-tazobactam was administered at 4.5 g every 6 h. Only the aerobic culture showed bacterial growth 38 h after starting culture. 16S rDNA sequence of this organism was difficult to determine because there were three strains with more than 99.9% homology with *N. macacae*, *N. sicca*, and *N. mucosa* with a difference in one, two, and three base values. Subsequently, the gram-negative diplococcus was identified as *N. macacae* based on MALDI-TOF MS analysis (Table 2) and biochemical characterization (nitrate test negative).

**Table 2:** Case 2: MALDI-TOF MS results

Rank	Matched Pattern	Score Value
1	*Neisseria macacae* DSM19175T	2.10
2	*Neisseria lactamica* CCUG26481	1.83
3	*Neisseria subflava* DSM17610T	1.81
4	*Neisseria mucosa* DSM17611T	1.78
5	*Neisseria lactamica* CCUG44970	1.77
6	*Neisseria enlogata ssp glycolytica* DSM23337T	1.77
7	*Neisseria lactamica* CCUG17628	1.77
8	*Neisseria subflava* CCUG29402	1.70
9	*Neisseria lactamica* CCUG796	1.70
10	*Neisseria lactamica* CCUG17016	1.70

No other organisms were isolated from the blood culture. Broth microdilution method (DP34, Eiken, Japan) showed that the bacterial culture was susceptible to ceftriaxone, ampicillin, meropenem, and minocycline but resistant to penicillin, trimethoprim-sulfamethoxazole, and levofloxacin (CLSI testing methods and MIC break point for *N. meningitidis* was substituted.). Transthoracic echography showed no vegetation. Fever had alleviated by day 4 of antimicrobial administration. Her treatment was changed to intravenous ceftriaxone at 2 g every 24 h. The total antibiotic treatment period was 13 days. Her condition improved, and she was discharged and followed up for 1 month in an outpatient setting, with no recurrence.

## Discussion and conclusions

*N. macacae* bacteremia is a rare disease, and its entry point and mechanism are often unknown. To our knowledge, there have been 10 specimens of *N. macacae* isolated in human samples, including these two cases (Table 3). There have only been two reports of *Neisseria* bacteremia in humans including a 5-month-old child and a healthy adult [1, 2]. This is the first report of two cases of *Neisseria* bacteremia in patients with cancer, one of whom had recovered from cancer. *N. macacae* is known to reside in the mouth of monkeys [3]; however, neither of our patients had a history of contact with monkeys. Only one case of infectious endocarditis in an adult with *N. macacae* bacteremia was reported [2].

**Table 3:** *N. macacae* isolated in human samples review

Case	Sex	Age (years)	Samples	References
1	F	42	Blood culture	Present case 1
2	F	69	Blood culture	Present case 2
3	M	0.66 (8 months)	Blood culture	[1]
4	M	65	Blood culture	[2]
5	F	52	Peritoneal fluid culture	[5]
6	NA	NA	Mouth	[4]
7	NA	NA	Throats swab	[4]
8	NA	NA	Respiratory tract	[4]
9	NA	NA	Respiratory tract	[4]
10	NA	NA	Respiratory tract	[4]

*NA* Not available

Previously, reports of *N. macacae* infection in humans include peritonitis in patients on peritoneal dialysis [5]. Addtionally, *N. macacae* has been isolated from the upper respiratory tract of neutropenic patients [4]. No neutropenia or no respiratory infections such as pneumonia and infective endocarditis were observed in our patients. In addition, echoendoscope colonization was reported, suggesting that it may have been established in the upper digestive tract of humans [6]. Among patients who used antibiotics within a month, *N. macacae* accounted for 4.5% of *Neisseria* species that colonized in the oral cavity [4]. Neither of our patients had used antibiotics within 1 month of admission, but both had previously been treated with antibiotics and chemotherapy. In both cases, mucosal damage was observed in the upper gastrointestinal tract. Therefore, exclusion diagnosis suggested that bacteremia invasion was caused by mucosal rupture in our cases.

There are no clear susceptibility testing methods or criteria for *N. macacae* detection, and the treatment for *Neisseria* bacteremia is unknown. Therefore, susceptibility testing was performed using the CLSI meningococcal criterion; however, it is unknown whether these results are appropriate. Both patients responded well to treatment with β-lactam antibiotics and improved after 2 weeks. The duration of treatment is unknown. Modifying the treatment based on the source of the infection may shorten the treatment period. Therefore, further research on *N. macacae* bacteremia is necessary.

Common *Neisseria* species that are pathogenic to humans include *N. gonorrhoeae* and *N. meningitidis*. 16S sequence analysis may not be sufficient to identify *Neisseria* species due to high intraspecies sequence variation in the 16S rRNA gene [4]. *Neisseria* species can be differentiated based on their morphological, physiological, and biochemical properties and MALDI-TOF MS results. Commensal *Neisseria* species that colonize oral and nasal cavity sites have only been rarely associated with disease [7]. Donati et al. have shown that commensal species have tropism for different sites in the oral cavity and oropharynx. *Neisseria meningitidis* is enriched for colonization in the throat, *N. flavescens* and *N. subflava* populate the tongue dorsum, and *N. sicca*, *N. mucosa,* and *N. elongata* the gingival plaque [8].

Interestingly, Bennett et al. have demonstrated that *N. subflava* and *N. flavescens* are phylogenetically the same species. Similarly, researchers have proposed that both *N. sicca* and *N. macacae* be reclassified as *N. mucosa* [9, 10].

The limitation of this report was that *Neisseria* spp. is difficult to fully identify with only 16sRNA. In 34% (29/85) cases of identification of the *Neisseria* spp., 16S rRNA sequence analysis indicated the possibility of more than one species [4]. Therefore, the usefulness of MALDI-TOF for the identification of *Neisseria* spp. is being examined. In CASE 2, the 16sRNA analysis results showed that *N. macacae*, *N. sicca* and *N. mucosa* were the three possible species. A combination of biochemical tests ruled out *N. mucosa* but did not differentiate N. macacae from N. *sica*. However, *N. sica* was not within rank 10 on the MALDI-TOF, and therefore *N. macacae* was identified.

This case report suggests that *N. macacae* bacteremia may be caused by gastrointestinal mucosal damage. Scrutiny of the upper gastrointestinal tract should be considered in patients with *N. macacae* bacteremia caused by an unknown source.

## Data Availability

Not applicable.

## Abbreviations

MALDI-TOF: Matrix-assisted laser desorption/ionization time-of-flight
MS: Mass spectrometry
CRP: C-reactive protein
CLSI: Clinical Laboratory Standards Institute
